# Flexible molecular chameleons: structural adaptability and therapeutic applications

**DOI:** 10.3389/fchem.2026.1886764

**Published:** 2026-08-18

**Authors:** Debosreeta Bose, Purvee Bhardwaj, Agnishwar Girigoswami

**Affiliations:** 1 Department of Basic Science and Humanities, Institute of Engineering and Management, University of Engineering and Management Kolkata, University Area, Kolkata, India; 2 JNCT Professional University, Bhopal, India; 3 Medical Bionanotechnology, Faculty of Allied Health Sciences, Chettinad Hospital & Research Institute (CHRI), Chettinad Academy of Research and Education (CARE), Chennai, India

**Keywords:** bioavailability, chameleon, flexibility, macrocyclic, permeability, simulation

## Abstract

There has been a growing focus on ‘molecular chameleons’ possessing structural flexibility; compounds that can dynamically adjust their conformations depending on the characteristics of the medium to either conceal or reveal polar parts in aqueous/lipidic environments. Drug discovery has shifted in the past few decades toward more complex molecules, such as cyclic and macrocyclic peptides, as well as PROteolysis-TArgeting Chimeras (PROTACs). These large macromolecules are intended to balance cell permeability, aqueous solubility, and strong target binding capacity for effective pharmacokinetics; though they are more difficult to design than conventional small drug molecules. Molecular chameleons are useful for this task because of their capacity to adapt to various environments. The science underlying the structural flexibility of molecular chameleons, their growing significance, role in therapeutics and design strategies using contemporary tools are all covered in this review.

## 1 Introduction

The term ‘Chameleon’ signifies mimicking or changing its properties for better applicative avenues. Molecular chameleons (MC) are unique compounds that can change their behaviour, such as how easily they dissolve or pass through barriers by adjusting their structure or the way they interact with their surroundings (Hoy, 1970). These flexible molecules can shift their shape and polarity in response to sudden environmental changes, where they play a major role as molecular probes. This ability makes them highly adaptable, especially when moving from a polar environment to a nonpolar one, which is quite promising in drug delivery and other pharmaceutical uses (Marjanović et al., 2019). An interesting observation where two apparently polar glucuronide metabolites, morphine 6-glucuronide and morphine 3-glucuronide, possessing lower lipophilicity than morphine itself, traverse the blood-brain barrier to activate the central analgesic mechanisms was reported by Carrupt et al. where they suggested that the molecules adopt a hydrophilic conformation in polar environments, whereas a lipophilic conformation with embedded or masked hydrophilic groups while crossing the biological blood-brain barrier (Carrupt et al.,1991).

In recent years, steering new and novel drug discovery drives with enhanced pharmacological properties, solubility, and permeability are underway, causing a paradigm revision of the conventional property-based drug design for greater clinical and therapeutic advancement (Caron et al., 2021; Jadach et al., 2024; Nyamba et al., 2024). In this regard, the unconventional candidates better labelled as *‘molecular chameleons’* are capable of undergoing conformational changes that expose polar groups in aqueous solutions but conceal them through non-bonded intramolecular interactions in nonpolar environmental settings, such as a lipid bilayer displaying both high aqueous solubility and high cell permeability promote itself to the new league of drugs (Furukawa et al., 2020; Mackman et al., 2018; Over et al., 2016; Rossi Sebastiano et al., 2018). Mackman et al. set an example with the discovery of an effective cyclophilin inhibitor having chameleonic characteristics with improved cell permeability and solubility for advanced clinical intervention (Mackman et al., 2018). Drug molecules with rigid conformations often exhibit limited permeability and adaptability to changing environments, consequently getting them trapped in the vicious cycle of balancing polar and nonpolar interactions within the same molecular arrangement. This limits their progression to meet the criteria for ‘drug’ candidates. This, in turn, increases their molecular weight by balancing their polar groups in contrast to non–polar substituents to increase aqueous solubility and hit their target (Whitty et al., 2016). Molecular chameleons can free themselves from such limiting conditions via the establishment of complementary intramolecular interactions among their polar functional groups while permeating a cell and then exposing them again inside the cell. Poongavanam et al. vividly substantiated the above mechanism through the molecular chameleon telithromycin, achieving aqueous solubility, cell permeability, and broad-spectrum activity (Poongavanam et al., 2024). Telithromycin, an orally administered antibiotic, principally adopts an open conformation in an aqueous solution, as evidenced by NMR studies. This substantiates the hydrogen bond formation between the polar groups of the molecule and water. In nonpolar environments, the molecule folds itself into closed conformations, allowing the formation of an intramolecular hydrogen bonding (IMHB) and an aryl-π–ketone interaction, reducing the polarity of the molecule for interactions encompassing biological lipidic environments. The conformational flexibility of chameleonic molecules allows them to bind to structurally analogous targets. Another similar macrocycle, roxithromycin, also adopts open and closed conformations in water and chloroform through the formation of IMHB rings and reduced polar surface area (PSA), promoting it to the category of molecular chameleons for enhanced drug efficacy (Danelius et al., 2020). Cyclic peptides encompassing such flexibilities in their structural conformations are also considered to fall under this category of molecular chameleons. These druglike compounds have been under the spectacles of medicinal scientists since the last decade, owing to their chameleonic properties (Guimarães et al., 2012).

Molecular chameleons are gaining a considerable edge among the scientific fraternity owing to the formulation of *beyond the Rule-of-5* (bRo5) therapeutics and high molecular weight drugs. Traditional drug targets, such as ion channels, enzymes, etc., can be modulated through low molecular weight drug molecules. However, some therapeutically viable targets still remain inaccessible by the conventional low molecular weight designed drugs. These challenging targets include protein-protein interactions, protein-nucleic acid interactions, complex protein scaffolds, dynamic interactions across bimolecular surfaces, etc. Thus, to address such complex binding targets more and more attention is being paid to design and develop bRo5 drugs. Recent decades have witnessed a fluttering evolution of such high molecular weight and novel drug moieties comprising macrocycles, cyclic peptides, proteolysis-targeting chimeras (PROTACS), molecular glues (MG), etc., which act as inhibitors in protein-protein interactions, modulates the functional aspects of certain proteins and even assist in their degradation (Arkin Michelle et al., 2014; Schreiber, 2021; Shultz, 2019; Valeur et al., 2017; Villar et al., 2014). MG induces the formation of ternary complexes by gluing proteins together, thus modulating their functions. Li et al. through their review article, emphasized CRBN (cereblon) to be involved in CRL4 (cullin 4-RING E3 ligase) complex degradation of MG targeted proteins. The complex E3 CRL4^CRBN^ ligase may be employed by the tiny-molecule MG of thalidomide, pomalidomide, avadomide, iberdomide, mezigdomide, eragidomide, etc. To ubiquitinate the MG-targeted protein neosubstrates of protein (Li et al., 2022).

Recent developments in drug discovery encompasses the integration of artificial intelligence (AI), *in silico* molecular docking, molecular dynamics simulations and structure–activity relationship (SAR) analysis. AI-driven approaches including machine learning, deep learning and generative AI models are exceedingly utilized nowadays for rational drug design and effective therapeutic intervention. This type of *in silico* approach limits the need of traditional trial-and-error based experiments with theoretical rationalization on virtual screening, targeted design, *de-novo* molecular design, digital–twin pharmacology, multi–omics data integration, and pharmacological and toxicological profiling (Mullowney et al., 2023; Zhang et al., 2025). Computational modelling has already demonstrated significant role in optimizing the discovery of novel kinase-based inhibitors through virtual screening, SAR based design of macrocyclic glutaminase inhibitors, anticancer targeting transporters, etc. (Qin et al., 2024; Ye et al., 2022; Xu et al., 2021). Integrating experimental results with theoretically computed values including X-ray crystallography, NMR analysis, cryo-electron microscopy, organoid design and screening, large phenotype data analysis provides a new Frontier for precision data driven drug discovery (Zhou et al., 2023; Zhu et al., 2025).

Thus, in the contemporary context of finding new generic medicinal compounds and drugs, study and research on molecular chameleons are worthy of their applicative genre. This current review emphasizes the identification of such flexible molecular chameleons and understanding their therapeutic potential in pharmaceutical and medicinal realms, thereby aiding researchers in their design and subsequent synthesis. We will comprehensively discuss the mechanistic impact of molecular chameleonic properties on the increased permeability and solubility of existing drug types and outline a probable design template for subsequent structure-activity relationship studies.

## 2 Modern drug design: physicochemical challenges and emergence of molecular chameleonicity

**FIGURE 1:**
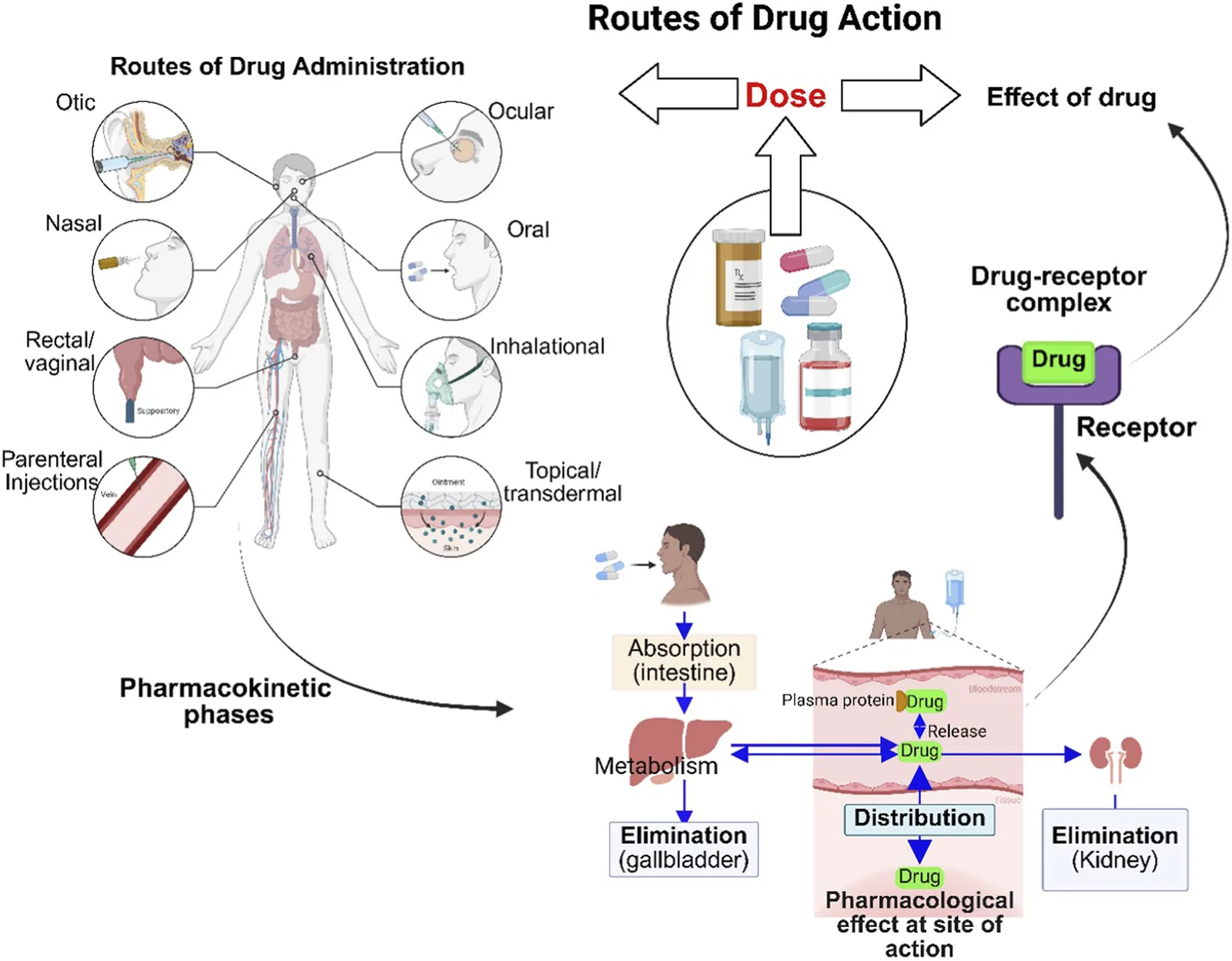
Figure depicting the mechanism of drug action within a living body (from dose to effect).

Successful drug development is parametricized as the combination of “drug target”, “drug action”, “drug modality” and “drug application” (Yamaguchi et al., 2021). An optimal balance between physicochemical characteristics of drug molecules determines their successful administration. Figure 1 comprehensively summarizes the overview of a drug action pathway, from successful administration to therapeutic outcome outlining the vital pharmacokinetic and pharmacodynamic characteristics (Testa and Mayer, 2003). Traditional drug molecules were formulated following the recommended guidelines given by Lipinski (Rule of 5) and Veber (Driggers et al., 2008; Hopkins and Groom, 2002; Lipinski et al., 1997; Veber et al., 2002). These empirical rules were particularly useful for low molecular weight drug molecules but were often found inadequate for large molecular weight compounds or drugs occupying ‘beyond the rule-of-5’ (bRo5) chemical space. Table 1 lists the empirical rules of Lipinski and Veber on the physicochemical characteristics of ideal drugs along with their implications on oral bioavailability are as follows.

**TABLE 1:** Lipinski’s and Veber’s rule for ideal drugs, along with their implications.

​	Parameter and rule	Implication
Lipinski’s rule of 5	Molecular weight (Da) ≤ 500	High molecular weight reduces permeability and affects its pharmacokinetic properties (absorption, metabolism, etc.)
	LogP ≤5	Measurement of lipophilicity, high values decrease solubility.
	Hydrogen bond donor (HBD) ≤ 5	Too many HBDs can hinder cell permeability
	Hydrogen bond acceptor (HBA) ≤ 10	Too many can reduce cell permeability
Veber’s rule	Low topological polar surface area (TPSA) ≤ 140	Affects bioavailability
	Low rotatable bond count (NRotB) ≤ 10	Affects bioavailability

**FIGURE 2:**
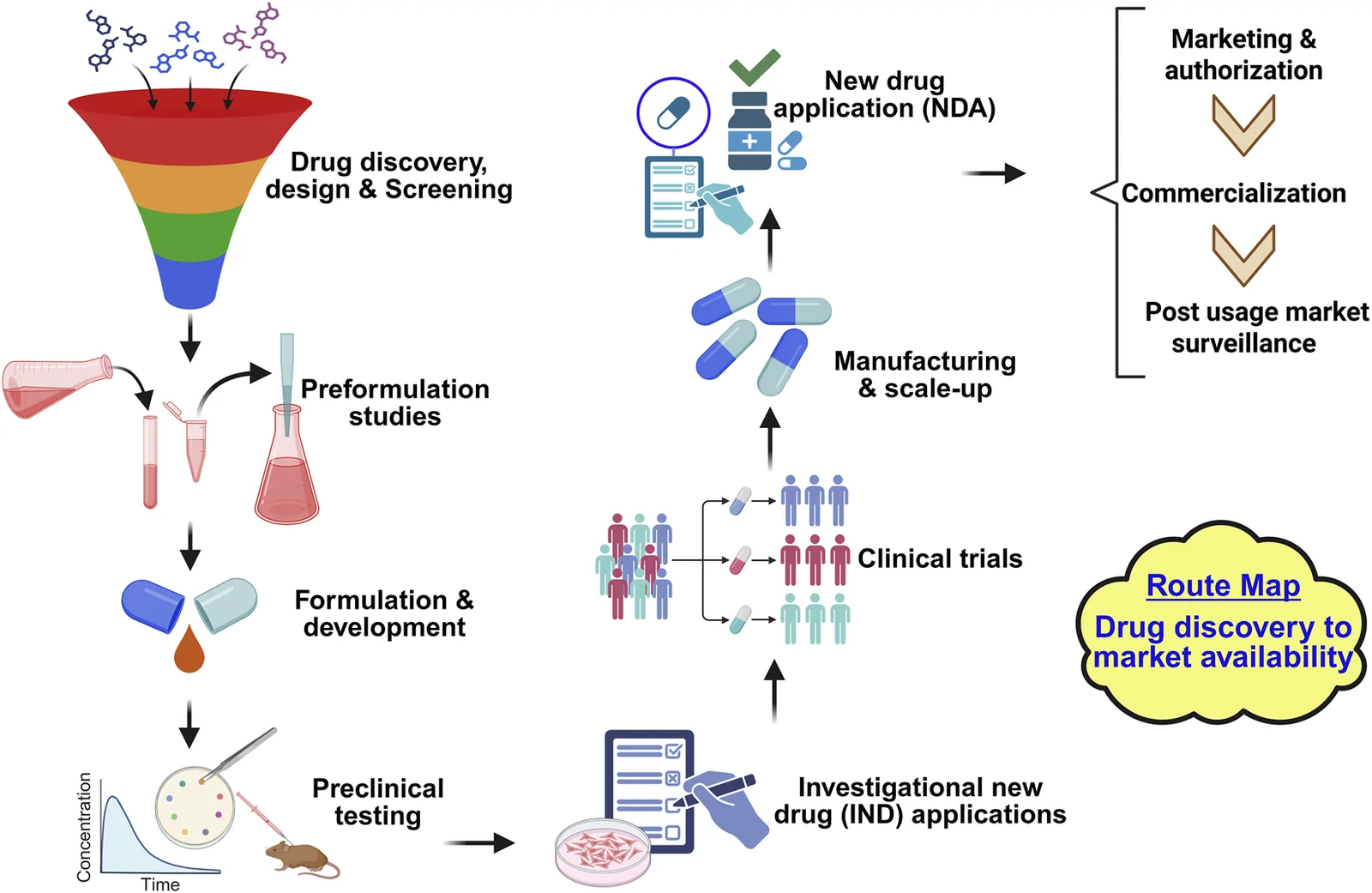
Figure depicting the route map from drug discovery to its availability in the market for clinical use.

Modern drug development aims to target “undruggable” disease pathways including complex biological targets, complex protein-protein interactions, allosteric binding pockets, intracellular interactions, targeted specificity, etc. Successful oral drug administration and bioavailability encompass a delicate balance between aqueous solubility, membrane permeability and metabolic stability. In this context, there has been an increasing interest on designing and developing high molecular weight and complex therapeutics capable of addressing the challenging “undruggable” targets. Thus, biologics, antibody–drug conjugate (ADC), molecular chameleons, macrocyclic compounds, cyclic peptides, PROTACs, etc., Are noteworthy classes of bRo5 drug molecules operating outside the empirical Ro5 chemical space (Bhalani et al., 2022; Chillistone and Hardman, 2017; Mantaj and Vllasaliu, 2020). Most of them are structurally complex, high molecular weight compounds possessing elevated polar surface area and having the provision of forming intramolecular hydrogen bonds, etc. (Doak et al., 2016; DeGoey and Cox, 2021). Figure 2 demonstrates a complete pathway of drug discovery, from design-to-development-to- successful market availability.

Beyond the Ro5 drug molecules often get trapped into balancing aqueous solubility and membrane permeability. Around 40% newly synthesized drug candidates often succumb to these ADME issues (Wu et al., 2024). Highly polar drug candidates possess enhanced solubility though its rates of diffusion are significantly reduced across cellular membranes (Matsson and Kihlberg, 2017). The physicochemical factors that influence *in-vitro* ADME properties along with the molecular descriptors used for their quantification are tabulated below (Table 2) (Jimenez et al., 2024a).

**TABLE 2:** Physicochemical properties and descriptors used in the bRo5 chemical space.

Ionization properties	Size and shape	Lipophilicity	Polarity	Chameleonicity
pKa in non-polar media	High molecular weight	BRlogD	E_PSA_	ChamelogD
pKa in water	Radius of gyration	logK_w_^IAM^	3D PSA	ChamelogK
​	​	cLogP	Δlog P_oct-dod_	​
​	​	ChromlogD	​	​

High molecular weight compounds possessing similar lipophilicity and topological polar surface area (TPSA) often differ in terms of membrane permeability pointing towards dynamic physicochemical properties to explain their behaviour (Pike et al., 2020). Hence, a considerable number of challenges exist that need to be addressed by drug developers or pharmaceutical scientists to improve the pharmacokinetic properties of drug molecules and formulations. Recent literature have comprehensively highlighted these challenges based on newly designed formulations and machine learning as predictive tool for understanding drug bioavailability (Morales et al., 2024; Vinarov et al., 2021; Zhang et al., 2024). Nucleic acid and protein-based drugs are the need of the hour, as evidenced by the 2024 Nobel Prize-winning research in Chemistry (Jumper et al., 2021; Kuhlman et al., 2003). However, substantial limitations and inadequate translational research remain in this sector to address the poor solubility and permeability limitations of new discovered drug candidates (Corbett et al., 2021).

Molecular chameleonicity emerges as a promising mechanism to explain the ‘druggable nature’ of certain bRo5 compounds. ‘Molecular Chameleons’ are often described as organic compounds possessing remarkable structural flexibility, with polar substituents exposed in polar solvents and formation of intramolecular bonds in non-polar solvents thereby reducing its overall polarity (Furukawa et al., 2020; Marjanović et al., 2019). They are explicit members of the bRo5 category of drug molecules for targeting complex substrates which traditional drug molecules cannot bind. The molecular characteristics of chameleonicity which significantly affect the properties of bRo5 drugs are explained in Table 3.

**TABLE 3:** Molecular features of chameleonicity and their effects on oral bioavailability in bRo5 drugs.

Molecular characteristics	Chameleonic mechanism	Effect on solubility	Effect on permeability	References
Intramolecular Hydrogen Bonding (IMHB) formation	Exposed non – polar groups in non – polar environments protecting the polar groups	Slight decrease in solubility	Significant increase in permeability	Danelius et al. (2020)
Conformational flexibility	Conformational adjustment with respect to environmental polarity	Aqueous solubility is increased	Facilitates diffusion processes across the cellular membrane	Ono et al., 2021
Reversible conformational switching	Open and closed conformations	Balanced aqueous solubility	Optimized membrane permeability	Inganäs et al. (2025)
Folded conformations	Decreases experimental polar surface area (EPSA)	Aqueous solubility is maintained	Facilitates passive diffusion across membranes	Jiménez et al. (2023)
Dynamic modulation of polar surface area (PSA)	Exposed heteroatoms with respect to environmental polarity	Increased aqueous solubility	Increased membrane permeability	Ahlbach et al. (2015)

The current review on molecular chameleons highlights an explicit class of bRo5 drug candidates with conformational adaptability to address the limitations of aqueous solubility and membrane permeability with targeted affinity, thus emerging as efficient therapeutic agents. Understanding these molecular mechanisms and evaluation of ‘molecular chameleonicity’ are inevitable for the rational designing of bRo5 drugs to expand the therapeutic framework in modern medicinal chemistry.

## 3 Physicochemical descriptors of chameleonicity

Molecular chameleons dynamically adjust their conformations with respect to environmental polarity in order to balance between aqueous solubility and membrane permeability. Small molecules typically conventional drug molecules mostly rely on static parameters to understand their physicochemical characteristics. Molecular chameleons on the contrary require dynamic physicochemical descriptors to account for their conformational flexibility, intramolecular interactions and polarity dependent structural adjustments. Thus, a number of quantitative descriptors are developed to understand the physicochemical basis and evaluate chameleonic scores in large complex molecules (Doak Bradley et al., 2014; Furukawa et al., 2020; Caron et al., 2021).

- Topological Polar Surface Area (TPSA): TPSA is a two-dimensional descriptor representing the total contributions from polar substituents (O, N, Cl etc.) in a molecule. TPSA is an important static descriptor for defining chameleonicity in molecules. Generally, TPSA is greater than 120–140 Å^2^ in molecules exhibiting chameleonicity. Classically, molecules with TPSA >140 Å^2^ do not favour membrane permeability though molecular chameleons are exceptions to this rule owing to their conformational adjustments based on solvent polarity (Fernandes and Gattass, 2009).
- Intramolecular Hydrogen Bonding (IMHB): IMHB refers to the formation of intramolecular hydrogen bonds between donor and acceptor moieties in the same molecule. IMHB is an important molecular descriptor in chameleonic molecules which allows the molecule to adopt a folded conformation in polar environments, masking the polar groups. This masking increases membrane permeability in molecular chameleons (Caron et al., 2021; Poongavanam et al., 2024).
- Experimental Polar Surface Area (EPSA): EPSA is an experimentally determined physicochemical descriptor of chameleonicity which reflects on the dynamic nature of polar functional groups present in a chameleonic molecule. Estimation of EPSA is experimentally conducted through supercritical fluid chromatography (SFC) in which the actual solvent exposed polarity and the flexibility effects of IMHB and folding can be understood in chameleonic molecules (Ermondi et al., 2020; Wang et al., 2024).
- Solvent Accessible Surface Area (SASA): SASA is the solvent accessible surface area typically water. It is a three-dimensional descriptor which provides a real picture of the exposed functional groups within the surrounding solvent. SASA is a dynamic descriptor quantifying the ratio between polar and non-polar surface area in chameleonic molecules (Jiménez et al., 2023; Poongavanam et al., 2024).

ΔPSA_3D_ or ΔSASA_polar_ Score = <SASA_polar,water_> - <SASA_polar,chloroform_>

The above equation refers to chameleonicity scores based on trajectory parameters and their performance is water v/s an organic solvent (generally octanol or chloroform).

v. *Radius of Gyration (R*_*g*_ *):* Radius of gyration is a structural parameter that describes molecular distribution around the centre of the compound. It represents a measure of molecular compactness and evaluates the propensity of a molecule to undergo structural adjustments with respect to solvent polarities (Doak Bradley et al., 2014; Poongavanam et al., 2024).
vi. *ΔEPSA:* The ease of conformational switching between two conformations in molecules with chameleonicity is measured via ΔEPSA. The value of ΔEPSA indicates how efficiently a molecule can switch between less folded polar conformations and less polar folded conformations in environments of varying polarities (Goetz et al., 2014).

A comprehensive table (Table 4) listing the various quantitative physicochemical descriptors for determining chameleonicity in molecules are as follows.

**TABLE 4:** Quantitative physicochemical descriptors for estimating molecular chameleonicity.

Descriptors	Typical quantitative range	Chameleonic mechanism	Examples	References
TPSA (Å^2^)	150 - 300	High TPSA is favourable for folded conformations increasing membrane permeability	ARV-110: 182.86 Cyclosporin: 229	Price et al., (2025); Jiménez et al. (2023); Jiménez et al., 2024b
EPSA (Å^2^)	90–100	Lower EPSA favours polarity masking	Cyclosporin: 120–140; Tacrolimus: 100–120	Poongavanam et al. (2018); Wang et al. (2024)
ΔEPSA (Å^2^)	20–80	High ΔEPSA value indicates high chameleonicity	PROTAC-110: 50–70; PROTAC-471: 50–70	Atilaw et al. (2021)
SASA (Å^2^)	Reduction of ∼30–70 Å^2^ in polar and non-polar solvents	High positive ΔPSA_3D_ or ΔSASA_polar_ signifies effective chameleonicity with deeply embedded polar substituents in non – polar solvents.	Sanglifehrin a ∼ 100 Å^2^ reduction	Villar et al. (2014)
Radius of gyration (R_g_) (Å)	5–15	Large shift in R_g_ values in solvents of varying polarities verifies structural chameleonicity	Sanglifehrin 8–10	Doak Bradley et al. (2014)

The energy barrier between two conformational states is another critical determinant to address chameleonicity in molecules. The difficulty in overcoming high potential energy barriers for cis–trans isomerization of peptide bonds through normal simulation protocols is a classic example of such phenomenon (Tang et al., 2023). A conformational energy barrier of ∼2–8 kcal/mol is often considered optimal for molecules showing efficient chameleonicity (Ermondi et al., 2023).

## 4 Influence of molecular chameleonicity on cellular uptake or permeability and water solubility

Molecular chameleonicity is particularly useful for high molecular weight drug molecules, especially those that fall beyond Lipinski’s rule of 5 (bRo5 drugs). These molecules can adopt flexible conformations with respect to environmental conditions. Their adaptability is crucial in enhancing cell permeability and maintaining aqueous solubility. It is well substantiated for orthodox druglike compounds that PSA ≤140 Å^2^ is typically a required parameter to have sufficient membrane permeability for good oral bioavailability. The three-dimensional structure of a compound determines the PSA of chameleonic compounds where polar atoms are buried or exposed to the surface of the molecules. Thus, quantitative methods such as determination of 2D-TPSA, 3D-PSA, EPSA, SASA, computational simulations thoroughly discussed in this review exist for calculating molecular PSA of high molecular weight and macrocyclic drugs. A PSA_aq_ > (0.2 × MW) and PSA_np_ ≤ 140 Å^2^ indicate the limiting parameters for existing molecular chameleons (Whitty et al., 2016). Strong hydrogen bonding interactions pertain to increased solubility of molecular chameleons in aqueous solvents. Cyclosporin A, a cyclic peptide and an immunosuppressant which exemplifies a potent prototypical molecular chameleonic bRo5 drug owing to its solvent-dependant conformational flexibility and ease of formation of IMHBs within the molecular system (Yang et al., 2023). It is a macrocycle capable of modulating its conformations in polar solvents thereby facilitating membrane permeability (Ono et al., 2021). Experimental approaches show the difference between values of the logarithmic partition coefficient in octanol and water (LogP_oct_), which is a measure to determine its lipophilicity. NMR experiments specifically Rotating-frame nuclear Overhauser Spectroscopy (NOESY/ROESY) studies in different solvent environments (polar/non-polar) reveal switching conformations between extended polar ones and compact shielded conformations. NOE/ROE further reveals long range interactions in CDCl_3_ solvents which is a characteristic for folded conformation in non-polar solvents. Presence of multiple IMHBs in the molecule stabilizes the folded conformations. These sorts of interactions are reduced in polar solvents making the molecule to remain in its extended non-folded conformation (Rüdisser et al., 2023). An energy barrier of ∼3–6 kcal/mol is required to be crossed for Cyclosporin A to rapidly switch between open and closed conformations (Ono et al., 2021). Anderson et al., in the late 1980s, showed the mechanism of action of vinylogous carbinolamine tumor inhibitors with chameleonic properties to overcome the low permeability issues associated with its polar surface molecules (Anderson and Mach, 1987). In another study, Engelberg et al. showed the advancements in epigenetic reader proteins, such as peptides and peptidomimetics, improve oral bioavailability via chameleonic behavior, modulating solubility and permeability dynamically. They can suitably be employed as novel therapeutic targets for a comprehensive range of diseases (Doak Bradley et al., 2014; Engelberg et al., 2021). Molecules with chameleonic properties can often be assessed through experimental techniques and observations like NMR analysis, HPLC measurements, and dynamic simulations like polar surface area (PSA) calculations to determine the impact on their working mechanisms as suitable drug targets.

**FIGURE 3:**
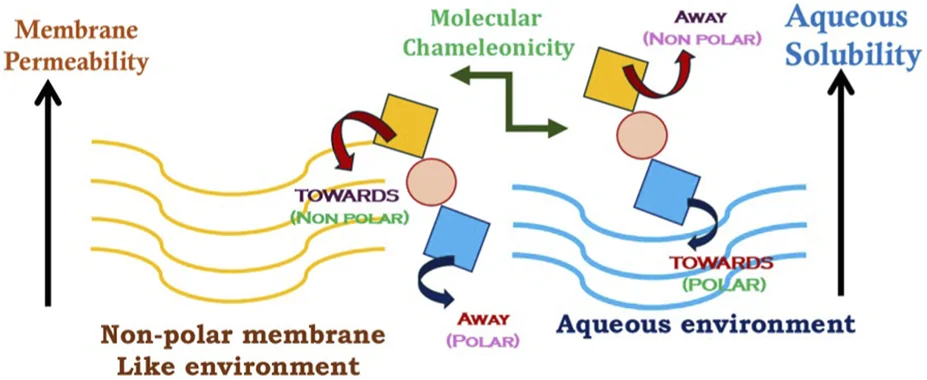
The change in orientations of molecular chameleons with respect to environmental polarity.

Drugs like Faldaprevir (non-macrocyclic) and Paritaprevir (macrocyclic) both bind to the smooth binding region of NS3/4 A protease inhibitor having TPSA values >140 Å^2^ in which molecular chameleonicity is argued to be the reason behind their high permeability (Sheikh et al., 2021). It is evident that the flexibility in conformation found in the molecular chameleons is responsible for their augmented bioavailability, which mainly originates from a synchronous intramolecular interaction within the molecule. A large number of orally bioavailable bRo5 drugs with molecular chameleonic properties are found to possess 5 to 15 rotatable bonds. This is in contrast to the other orally bioavailable drugs with molecular weight <500 Da, whose sum total of rotatable bonds is negligibly less. The presence of rotatable bonds in these drug molecules is a useful indicator of molecular flexibility and chameleonicity in the case of non-cyclic moieties (Sheikh et al., 2021). Cyclic moieties or ring systems can also include molecular chameleonic behavior, where the flexibility index is monitored by the Kier flexibility index (Φ) (Caron et al., 2020). A higher Φ value indicates greater conformational flexibility facilitating the switching between open and closed molecular conformations indicating the existence of molecular chameleonicity. Hence, static physicochemical descriptors as TPSA cannot solely define chameleonicity in certain molecules but dynamic conformational descriptors are also taken into consideration. The changes in orientation of molecular chameleons in water and membrane can be visualized through Figure 3. This figure provides a comprehensive visual aid of the working mechanism of molecular chameleons.

## 5 Different classes of molecular chameleons

Molecular chameleons are well known for their versatility in terms of structure and function. Their dynamicity and wide functional horizon make them the most coveted molecules in drug development and discovery. The structural complexity of molecular chameleons is quite interesting owing to their classification in terms of small molecules and macro structured compounds. At the molecular level *‘molecular chameleons’* can be classified as *(i) small chameleonic molecules* and *(ii) macrocyclic chameleons*. Small drug molecules with chameleonic properties as specific sulphone drugs fall under the category of *“small chameleonic molecules”*. Macrocyclic chameleons include macrocyclic natural products, cyclic peptides, macrocycle derivatives, etc. PROTACs (Proteolysis-Targeting Chimeras) are a class of functional molecular chameleons, they are essentially bRo5 therapeutics with high molecular weight encompassing substantial membrane permeability. Supramolecular carrier systems with chameleonic characteristics and employed as smart drug delivery systems are referred to as *‘chameleonic carriers’* (Negut and Bita, 2023; Zhang et al., 2022). Here, in this section we highlight the design and structure-activity relationship analysis of small molecular chameleons (sulphone-based chameleonic drugs), macrocyclic chameleons (peptides) and PROTACs working as ‘molecular chameleons'.

### 5.1 Sulfones and similar molecules as chameleon

**FIGURE 4:**
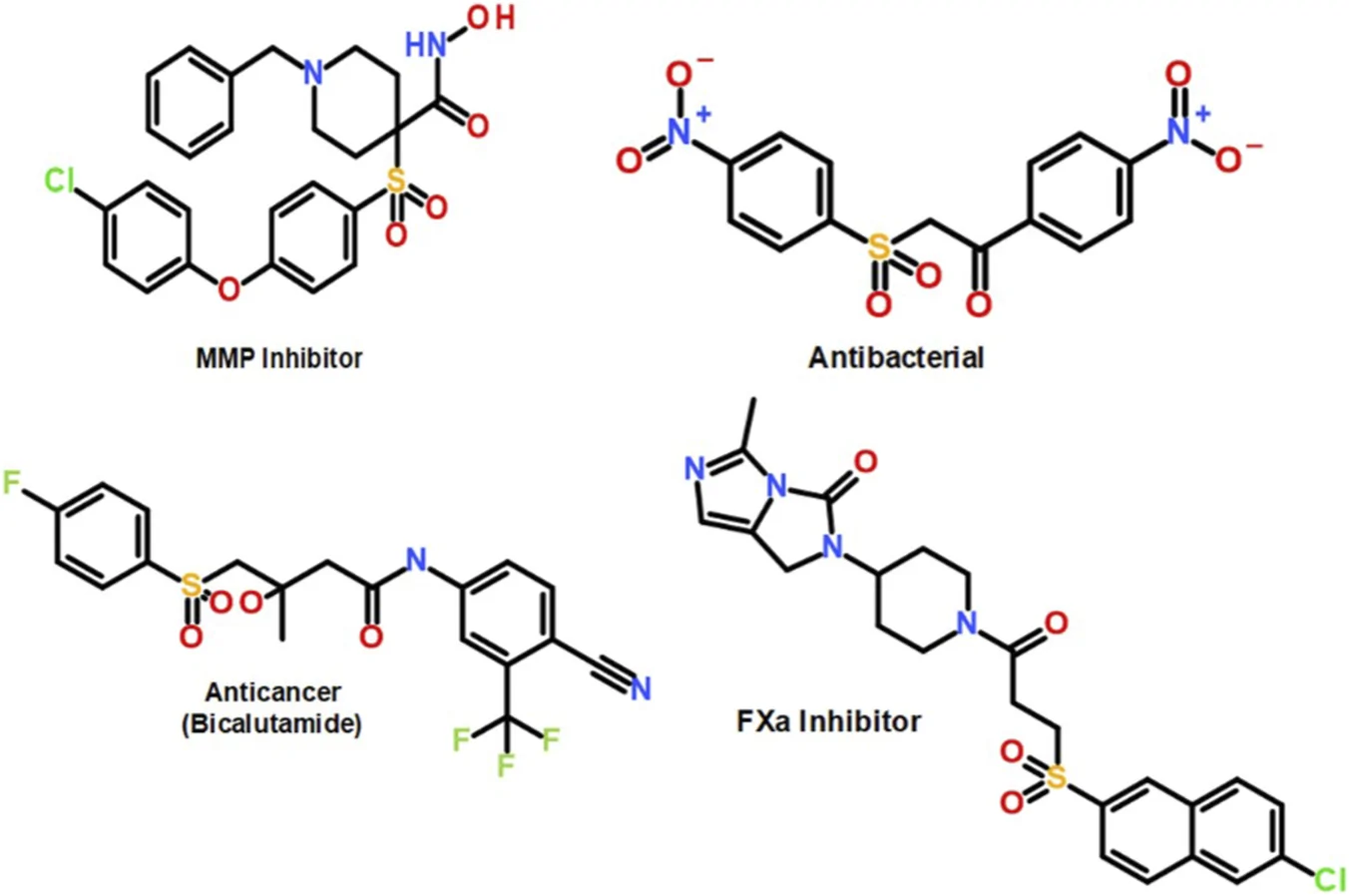
Typical bioactive keto sulphone derivatives. (Reprinted (adapted) with permission from Green Chemistry, 2023, 25, 8273–8279. Copyright 2023, Royal Society of Chemistry) (Xia et al., 2023).

The term “molecular chameleon” represents the class of compounds which can dynamically adjust their conformation depending on environmental conditions and adapt accordingly to score a balance between aqueous solubility and membrane permeability. In this context *molecular chameleons* are distinctly different from *chemical chameleons*. From the purview of synthetic organic chemistry, chemical chameleons are compounds that exhibit chemical diversity and participate in multiple reaction pathways. Sulfones are often referred to as *“chemical chameleons,”* owing to its versatile electron withdrawing functionality. It has a propensity to bind with biological targets drawing a balance between aqueous solubility and membrane permeability, the primary attribute of molecular chameleons. Barry et al. in their review, described molecules with sulphone functionalization as chemical equivalents of small molecular synthons (Trost and Kalnmals, 2019; Chu et al., 2015; Hok and Schore, 2006). In the realms of drug development, sulfone drugs are widely used for their antibacterial and anti-inflammatory properties. Specially designed molecular sulphone derivatives can exhibit chameleonic properties with regulated polar surface area and intermolecular interactions making them important therapeutic candidates in medicinal chemistry. Vinyl sulfones, in this context, are at the forefront of drug discovery and development due to their ubiquitous skeleton, which occurs in many natural products (Tong et al., 2024; Breuer et al., 2018). α,β-unsaturated phenyl vinyl sulfone has garnered considerable attention as a cysteine protease inhibitor (Xia et al., 2023). β-carbonyl sulfone derivatives and γ-keto sulfones are well reported to combat bacterial infections. The structural representation of some sulphone derivatives listed in this category is shown in Figure 4 below (Xia et al., 2023).

The notable sulfone-based drugs with versatile properties mirroring the chameleonic properties may be tabulated as follows (Table 5).

**TABLE 5:** Sulphone drugs highlighting their therapeutic application and roles.

Sl. No.	Drug name	Therapeutic use	Role of sulfone group	Mechanistic role	Chameleonic characteristic/Contribution	References
1	Furosemide	Diuretic	Sulfone group enhances diuretic activity by inhibiting sodium and chloride reabsorption	Sulfonamide moiety binds to K^+^/Ca^+^/Cl^−^ co-transporters	Conformational flexibility required for pharmacological action.	Thakuria et al. (2013)
2	Sulfasalazine	Rheumatoid arthritis, ulcerative colitis, Crohn’s disease	Sulfone moiety contributes to anti-inflammatory effects	Sulfonamide moiety in sulfasalazine inhibits NF-κB and modulates inflammatory cytokines.	Undergoes amide-imide tautomerism providing conformational flexibility to achieve chameleonic character and functionality.	Tahir et al., 2021; Jia et al., 2021
3	Darunavir	Antiretroviral protease inhibitor for HIV infection	Sulfone group enhances binding affinity to HIV protease, improving efficacy	-O Groups forms bis-hydrogen bonds with Asp30 of HIV proteases stabilizing the inhibitor-enzyme complex.	Bis-tetrahydrofuran group undergoes conformational switching to behave as chameleons both structurally and functionally	Lascar and Benn (2009)
4	Sumatriptan	Migraines, headaches	Sulfone group aids in selective serotonin receptor agonism	Sulfonamide groups in sumatriptan optimizes the H-bond donor/acceptor geometry for 5-ht1B/1D receptor activation.	No conformational switching but structural alignment of hydrogen bond donor/acceptor for targeted binding	Group (1991)
5	Bosentan	Pulmonary arterial hypertension	Sulfone moiety contributes to dual endothelin receptor antagonism	Sulfonamide groups in bosentan helps in maintaining a -ve charge at physiological pH to interact with the specific endothelial receptors.	Undergoes bond rotation connecting its central pyridine ring and outer bonds. Capable of structural adjustments with respect to environmental polarity	Montero-Pérez-Barquero and Morales-Rull (2016)
6	Glimepiride	Type 2 diabetes mellitus	Sulfone group aids in stimulating insulin release from pancreatic β-cells	It triggers depolarization of the ATP sensitive K^+^ channels. Sulphonyl moieties of glimeperide reorients the molecule and acts as a molecular linker.	Conformational adjustments with respect to environments.	Briscoe et al. (2010)

These examples highlight the versatile nature of some specific drugs with sulphone functionality which contributes to conformational adaptability, environmental adjustability, hydrogen bonding interactions, etc., Though not applicable to all sulphone-based derivatives. Apart from being considered as ‘chemical chameleons’ these sulphone molecules also adapt to environmental conditions with adjustable conformations underscoring a balance between solubility, permeability, targeted binding and pharmacological efficiency. Thus, the physicochemical attributes of some specialized sulphone architectures induce chameleonicity making them instrumental molecules in therapeutics.

### 5.2 Macrocyclic natural products

**FIGURE 5:**
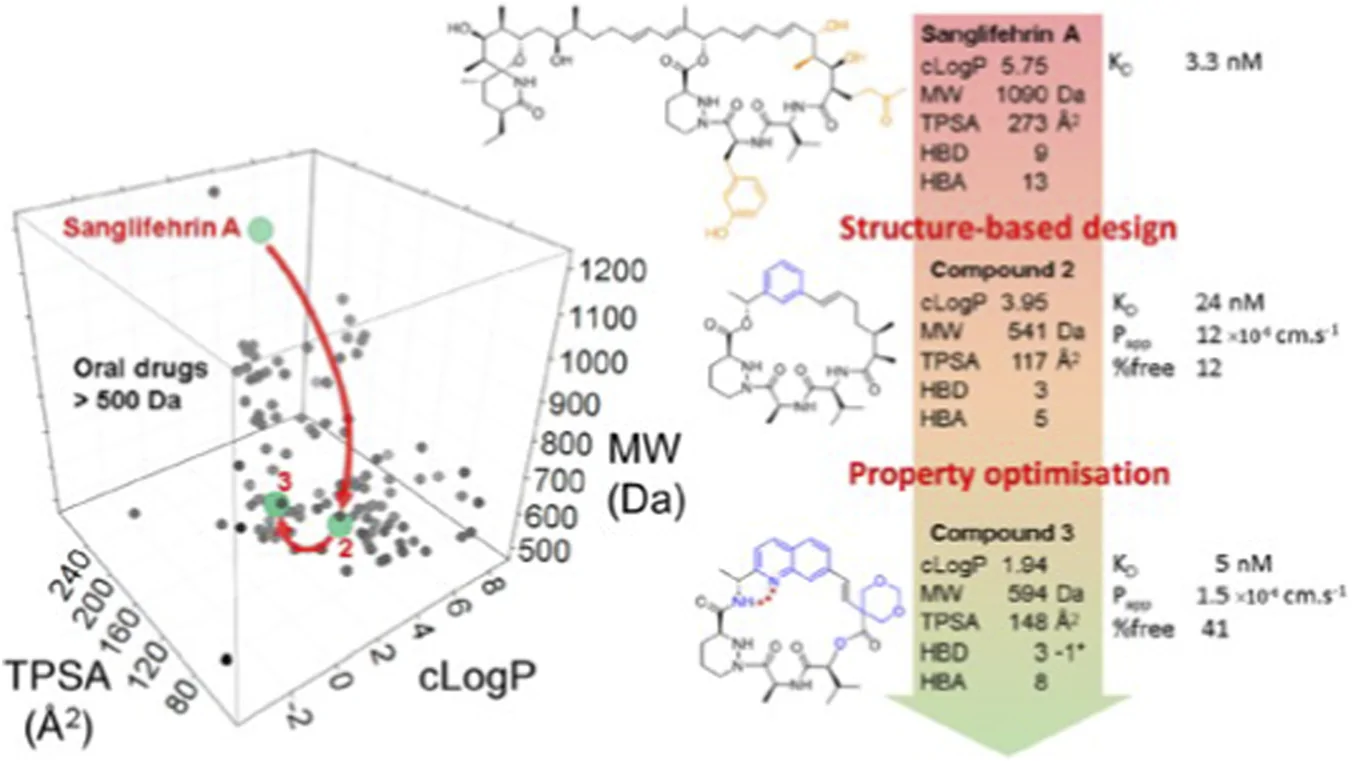
The overall track for the development of sanglifehrin A into druglike analogs 2 and 3 encompassed a strategic, structure–activity-based optimization that inhibit cyclophilin A. Computed physicochemical characteristics and experimentally determined in vitro properties of the above cited compounds are given adjacent to the structures. In vitro properties provide signatures of inhibition of binding of a FRET probe to cyclophilin A (K_D_), permeability across Caco-2 cell monolayers in the A → B direction (P_app_), and % free in human plasma (% free). The 3D cube shows the preparatory point of sanglifehrin A, key compounds 2 and 3, as well as oral drugs having MW > 500 Da (gray dots) in TPSA, cLogP, and MW space *("Reprinted (adapted) with permission from J. Med. Chem. 2018, 61, 9469–9472. Copyright 2018, American Chemical Society")* (Doak et al., 2018).

Macrocyclic natural products, specifically encompassing beyond the *rule of 5* chemical space, are known to exhibit ‘chameleonic behavior.’ Groove-shaped binding sites in biological targets like cyclophilin are difficult to bind with small drug molecules. Doak et al. comprehensively demonstrated the rescue of such targets through macrocyclic natural products, illustrating the example of sanglifehrin A, which ultimately got transformed into an orally bioavailable potential drug (Doak Bradley and Kihlberg, 2018). Sanglifehrin A can be structurally modified to a macrocycle to produce an inhibitor of cyclophilin A. The structural modification posed the potency to alleviate the oral bioavailability of the drug through the formation of IMHB and inhibiting the plasma-protein binding. The structure-based design improvisations depicting the property optimizations of the structurally modulated derivatives of Sangliferin A were beautifully depicted by Doak Bradley and Kihlberg (2018). The figure may be illustrated as follows (Figure 5).

Computed physicochemical characteristics and experimentally determined *in vitro* properties of the above cited compounds are given adjacent to the structures. *In vitro* properties provide signatures of inhibition of binding of a FRET probe to cyclophilin A (K_D_), permeability across Caco-2 cell monolayers in the A → B direction (P_app_), and % free in human plasma (% free). The 3D cube shows the preparatory point of sanglifehrin A, key compounds 2 and 3, as well as oral drugs having MW > 500 Da (gray dots) in TPSA, cLogP, and MW space (“Reprinted (adapted) with permission from J. Med. Chem. 2018, 61, 9469−9472. Copyright 2018, American Chemical Society”) (Doak Bradley and Kihlberg, 2018).

**FIGURE 6:**
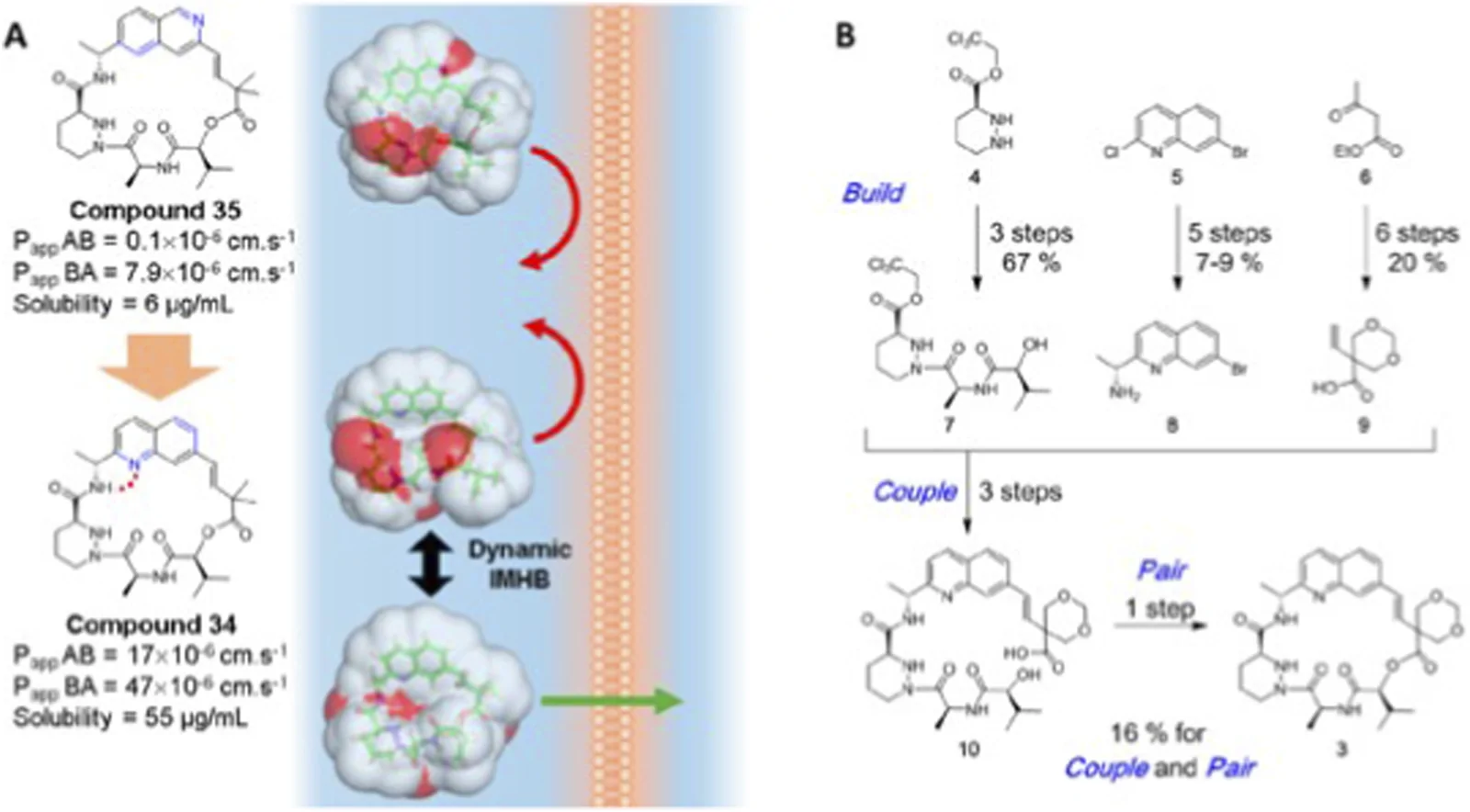
**(A)** The formation of a dynamic IMHB can be apprehended from the design of compound 34 from 35, which might be the reason behind the chameleonic character of 34. Has The calculated 3D polar surface area (PSA) in Compound 35 is of the value 132 Å^2^, whereas chameleon 34 can accommodate conformations for which the 3D PSA varies between 133 and 92 Å^2^ because of the same IMHB dynamicity. For illustrative purposes the conformers and 3D PSA were calculated. The solvent-accessible surface area encompassing the 3D PSA of the heteroatoms N, O, and attached H atoms, were constructed. The colour notations signify red (polar), while white the nonpolar surfaces in the structures. Cell permeability was estimated across monolayers of Caco-2 cell as reported by Mackman et al. (2018). **(B)** A convergent synthetic route for the preparation of candidate drug 3 and analogs are outline and developed. Compound numbers as denoted above are those used by Mackman et al. (“Reprinted (adapted) with permission from J. Med. Chem. 2018, 61, 9469−9472. Copyright 2018, American Chemical Society”) (Doak Bradley and Kihlberg, 2018).

The formation of IMHBs in the structurally modified derivatives is important in terms of the synthesis pedagogy to affect such modifications, ultimately increasing their drug potency. The figure, as shown in their article by Doak et al., can be presented as Figure 6.

**FIGURE 7:**
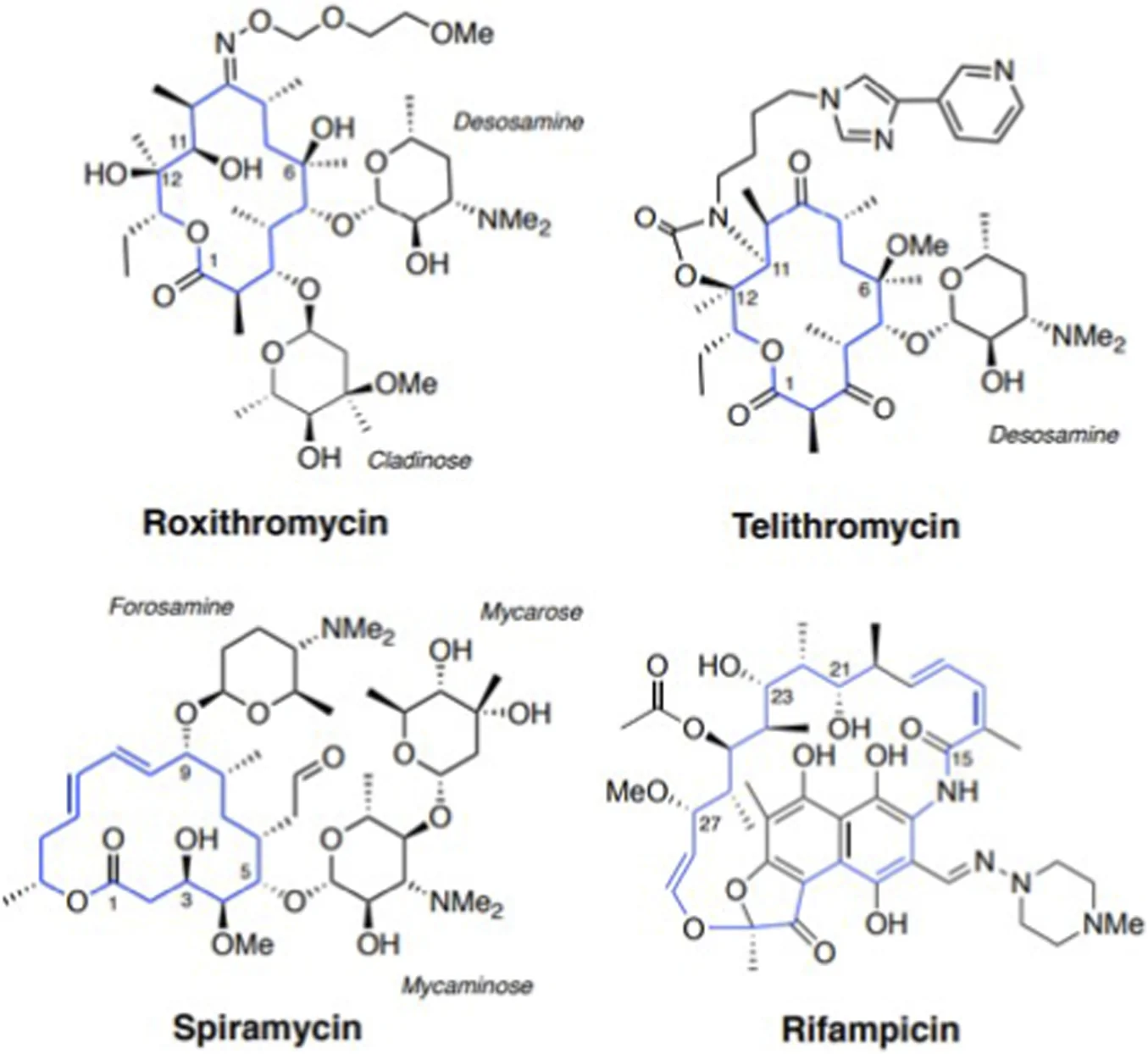
Structures of the four macrocyclic drugs (Roxithromycin, Telithromycin, Spiramycin, Rifampicin) for which conformational ensembles have been determined in CDCl_3_ and D_2_O. The macrocyclic core of each drug is shown in blue color, and the names of the attached saccharides are in italics. (Reprinted (adapted) with permission from Chemistry: A European Journal, 2020, 26, 5231−5244. Copyright 2020, John Wiley and Sons and Copyright Clearance Center”) (Danelius et al., 2020).

Danelius et al. (2020) presented the four macrocyclic drugs—roxithromycin, telithromycin, spiramycin, and rifamycin—with their underlying conformational assemblies, which display saccharides in their structures for D_2_O and CDCl_3_ solubility. This is shown below in Figure 7 (Danelius et al., 2020). The study highlights the importance of conformational flexibility in bRo5 drugs to determine chameleonicity. Several physicochemical parameters other than molecular weight determines the chameleonic properties in drug molecules. Among the four compounds investigated by Danelius et al. rifamycin revealed more chameleonicity owing to its polarity masking compared to the other macrolides. Dynamic descriptors are more effective than static descriptors for assessing molecular chameleonicity (Danielius et al., 2020).

### 5.3 Peptides

**FIGURE 8:**
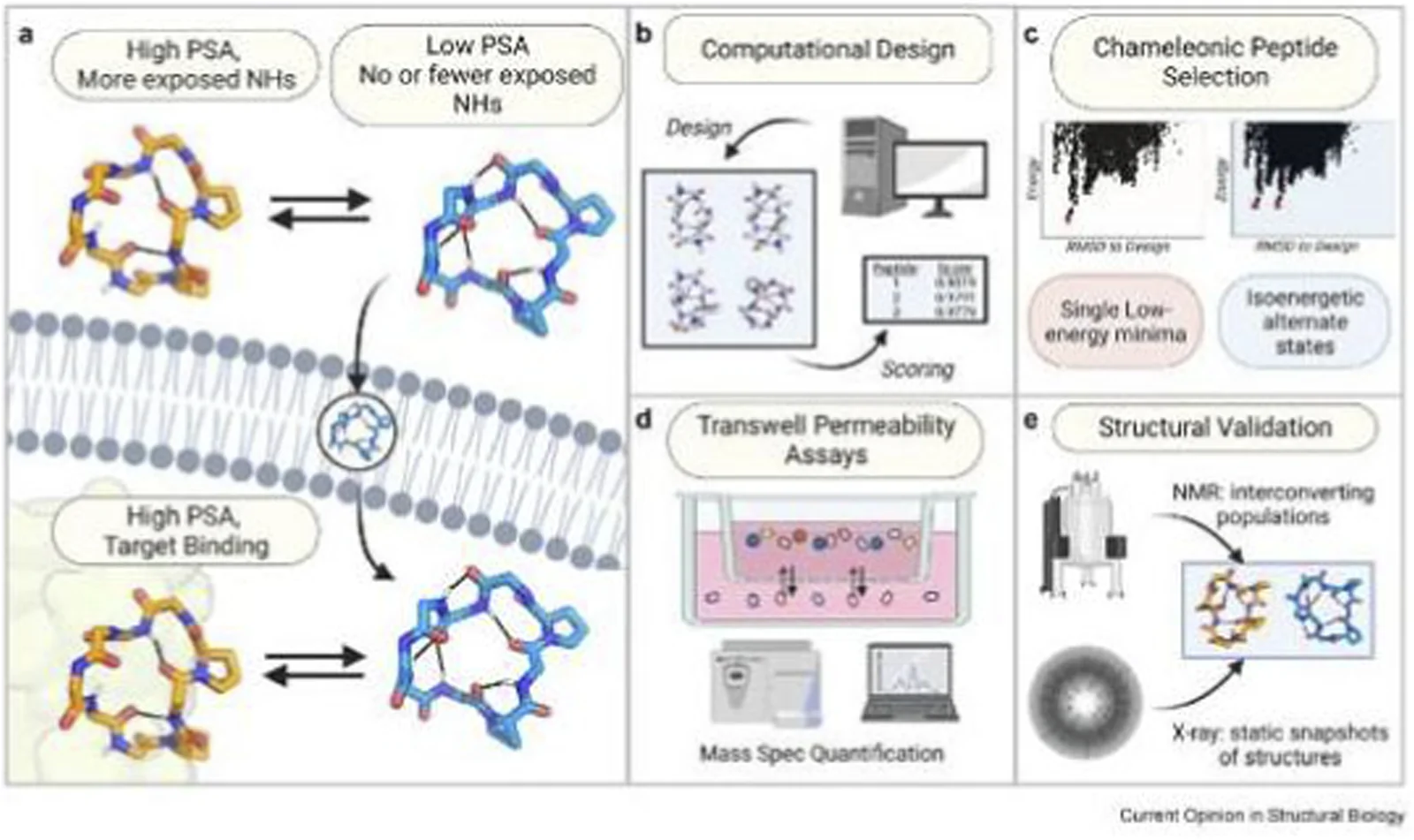
A graphic computational methodology for designing Chameleonic Peptides: **(a)** Proposed mechanism for passive permeability in chameleonic peptides. Conformational equilibrium is existent in majority of macrocyclic peptides. When in assembly with a hydrophobic cell membrane, the shift in equilibrium is towards the lowest PSA conformation, offering passive permeability. **(b)** A library of macrocyclic peptides are computationally designed generating thousands, and sometimes millions, of designs. This library of designs is filtered by scoring metrics. **(c)** Energy prediction landscapes assist in the identification of chameleonic peptides. The traditional potential energy curve (PEC) calls for a “funnel” shape, resulting in a single low energy minimum. The chameleon PEC outlooks two or more deep energy minima. **(d)** Transwell assays provide *in vitro* data on permeability. Peptides added to the donor are well diffused across the membrane and into the acceptor well. Mass spectrometry estimates permeability with the amount of crossing. **(e)** Macrocyclic peptides that demonstrate permeability are structurally validated using NMR and X-ray crystallography. NMR is capable of capturing the signals of multiple peptide conformations as they interconvert in solution.

Peptides or short-chain amino acids are miniature versions of proteins. Chameleonic peptides mainly encompass alterations in the sequence of amino acids within their secondary structures, i.e., adopted α helices and β sheets. Peptides with cyclic structures can effectively switch between different conformations owing to their segmental flexibility, which increases their oral bioavailability as drug molecules (Linker et al., 2022; Whitty et al., 2016). Generally, low PSA-valued peptides can show improved passive permeability. The formation of IMHBs contributes to such parameters. Ramelot et al. (Ramelot et al., 2023) effectively designed chameleonic peptides with high cell permeability exploiting molecular dynamics (MD) simulation. A *de novo* peptide design and development suggests improvements in organizational configurations of such peptides to elevate their drug efficacy. Ramelot et al. designed a number of membrane-traversing macrocycles containing 6–12 amino acids including N-methylated ones, a subset of which were designed to alter conformations in a solvent-dependent manner. Their computational design strategy is shown below in Figure 8 (Ramelot et al., 2023).

**FIGURE 9:**
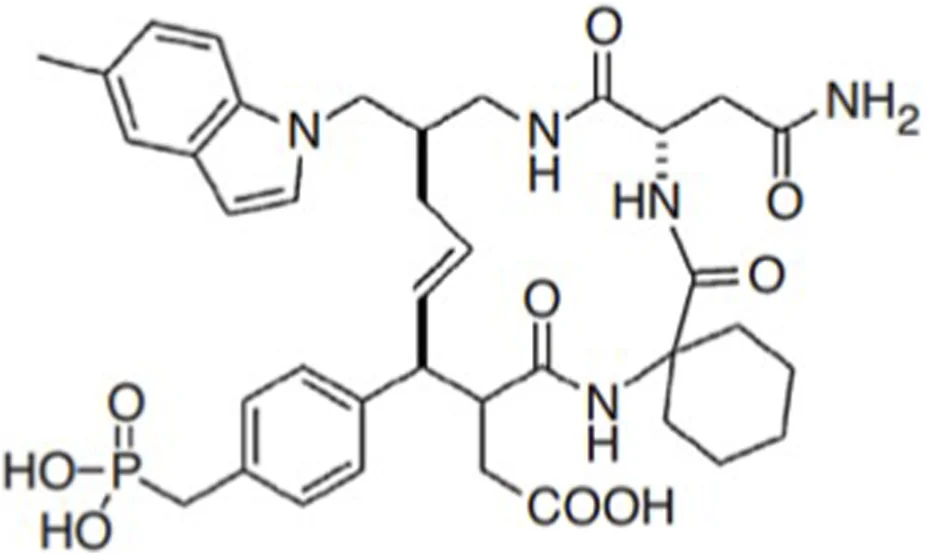
Pictorial representation of generated Grb2 SH2 domain binding ligand.

Solvents can influence the chemical structures of peptides by shifting the equilibrium towards one conformation or another. Static complementary conformational images of chameleonic peptides are captured through crystallographic investigations (Ramelot et al., 2023). These chameleonic peptides target intracellular substrates, particularly encompassing protein-protein interactions (PPI), thus widening the vertical of their applications as drugs. Qian et al. elaborated structure-based designs for generating cyclic peptides as PPI inhibitors in the following manner (Qian et al., 2017):

- Stapled α−helical peptides or hydrocarbon stapled peptide helices.
- β−Turn mimetics like Grb2 SH2 domain binding ligands are shown below in Figure 9
- Peptide loops are stapled together.

In addition, they also integrated biologically and chemically synthesized libraries for combinatorial peptide designing methodologies. Among these, chemically synthesized libraries, such as one-bead-two-compound libraries with cyclic cell-permeating peptides (CPPs) (Jia et al., 2021), DNA-augmented or templated macrocycles or peptides (Lascar and Benn, 2009), and the synthesis of targeted organic ligands (Group, 1991) can be fused together.

Several methodologies were highlighted by Qian et al. for enhancing membrane permeability. N^α^−methylations of cyclic peptides, formation of IMHBs, and hydrophobic property disseminations within the peptides are the prime chemical road maps for inducing chameleonic properties within cyclic peptides (Ahlbach et al., 2015; Qian et al., 2017; White et al., 2011).

Simple reduction of molecular weights in macrocyclic peptides often do not suffice the drugability parameters for successful therapeutic interventions. Engineering conformational flexibility clubbed with wide range polarity distributions for better metabolic adjustments are currently the emerging viewpoints for better drug discovery. In this context, Liang et al. developed c10c-G1V3, an optimized peptide from the membrane permeable Tat-GluA2-3Y peptide through chameleonic optimization strategies. The therapeutic limitation of Tat-GluA2-3Y was overcome by its backbone N-methylation and macrocyclization improving plasma stability and pharmacokinetic ability. As discussed, earlier N-methylation is a successful chemical modification strategy to induce chameleonic properties in a molecule facilitating compactness and reduction of PSA (Bockus et al., 2015). Cyclization on the other hand induces a trade-off between conformational flexibility and entropic surge resulting in increased cellular permeability and decreased degradation (Liang et al., 2026). Another classic example of drugability enhancement in cyclic peptides was provided by the optimization of cyclin A/B inhibitors. Protein–protein interactions are classically difficult interaction strategies owing to limited permeability. These cyclin RxL A/B inhibitors through iterative optimization emerged as passive permeable molecules with high oral bioavailability and clinical applications (Shapiro et al., 2026). In a recent article Josien and co-workers discovered enlicitide (MK-0616) through a late-stage optimization protocol referred to as ‘Design-Make-Test-Analyze (DMTA)’ to induce chamelonicity in a peptide molecule for preclinical profiling. Several bottlenecks like, poor aqueous solubility, complex synthesis procedure, metabolic instability in the initial optimization protocols were overcome through strategic modifications of the molecule via a re-engineered macrocyclic cross-link. Introduction of polar motifs into the molecule design increased its aqueous solubility while preserving its pharmacokinetic ability (Josien et al., 2026). The aryl-Daptomycin derivative developed by Yan et al. through post-modification of the tryptophan residues improved its antimicrobial potency, antibiofilm activity and membrane stability. Hydrophobic chain engineering was the primary highlight of the article which finally induced chameleonic properties in the molecule augmenting its therapeutic potential (Yan et al., 2026). Thus, these scaffold engineering strategies based on chemical modifications (N-methylation, cyclization, etc.), hydrophobic chain modification, conformational iterations, tuning solvent polarity better optimizes the macrocyclic peptides for therapeutic applicability. Thus, introducing chameleonic properties within these molecules eases a sound balance between aqueous solubility, membrane permeability, increased stability and therapeutic efficiency beyond the paradigms of conventional bRo5 drugs.

Table 6 represents a collection of some well-known chameleonic drug molecules and their associated physicochemical parameters for effective bioavailability.

**TABLE 6:** Reported physicochemical characteristics of some well-known molecular chameleonic drugs.

Name of the compound	Class	Molecular weight (Da)	TPSA (Å^2^)	EPSA (Å^2^)	Hydrogen bonding (Donor/Acceptor)	Key chameleonic characteristic	References
Cyclosporin A	Cyclic peptide	∼1200	280	∼120–140	5/12	Capable of forming IMHBs and possess conformational flexibility	Ono et al. (2021)
Telithromycin	Ketolide	815	190	∼110–140	2/14	Conformational flexibility and closed conformation in polar solvents	Price et al., (2025)
Simeprevir	Macrocycle	750	165	∼90	3/10	Shielded polar groups	Wieske et al. (2023)
Faldaprevir	Non-macrocycle	870	150	∼90–120	2/11	Conformational adaptation based on solvent polarity and formation of IMHBs	Jiménez et al. (2024)
Paritaprevir	Macrocycle	765	200	∼100–130	2/10	Conformational flexibility and masked conformation in polar environments	Sheikh et al. (2021)
Rifampicin	Macrocycle	822	220	∼140–160	6/16	Conformational adjustment based on solvent polarity	Ermondi et al. (2021)
Sanglifehrin A	Macrocycle	1170	290	190–220	6/15	Conformational flexibility and exposed IMHBs	Doak Bradley and Kihlberg (2018)
ARV-110 PROTAC	PROTAC	810	185	∼110–140	4/15	Conformational folding reduces exposed polar surface area	Nguyen et al., (2022)
ARV-471 PROTAC	PROTAC	1050	240	∼130–160	5/19	Dynamic conformational adaptation and folded structure in polar environments	He et al. (2025)

### 5.4 General design principles for inducing chameleonicity in a molecule

Molecular Chameleons encompass a diverse class of conformationally flexible molecules drawing a subtle balance between aqueous solubility and membrane permeability. Designing a chameleonic molecule incorporate some common design principles which aid in controlling a dynamic balance between aqueous solubility and membrane permeability underscoring their enhanced efficacy in therapeutics. Their chameleonic dynamicity is mainly induced through structural design principles with adjustable conformations as discussed.

#### 5.4.1 Engineered IMHBs in a molecule

Strategically placing donor and acceptor moieties in a molecule so as to form intramolecular hydrogen bonding within it specially in polar environments can induce chameleonic properties within the molecule. Jimenez et al. in their recent article highlighted IMHB mediated chameleonicity in CRBN-based PROTACs targeting BET (Bromodomain and Extra-Terminal) proteins (Jimenez et al., 2024a). This is a classic example of tuning a molecular architecture towards forming IMHBs to induce functional chameleonicity in a molecule. Intramolecular proton transfer within the same molecule enabling keto-enol tautomerism are also excellent methods to engineer IMHB in a molecule (Bose et al., 2024; Sil et al., 2023).

A classic example of such tautomerism directed molecular chameleonicity is shown by the antiviral elbasvir, a Hepatitis C virus NS5A inhibitor. In this particular molecule the hydrogen bond donor and acceptor functionalities are influenced by keto-enol tautomerism. Keto-enol tautomerism in this compound alters the IMHB patterns inducing a conformational flexibility in this molecule relative to the environmental conditions (Wieske et al., 2023).

#### 5.4.2 Inducing controlled flexibility in a molecule

Chameleonic molecules must possess a subtle balance between flexibility and rigidity in their structures. Highly flexible molecular architecture limits its target binding capacity increasing the entropic payload whereas excessive rigidity makes the molecule less adjustable to changing environmental conditions. Caron and co-workers comprehensively described the physicochemical descriptors that makes a molecule chameleonic and design strategies to discover bRo5 drugs (Jimenez et al., 2024b).

#### 5.4.3 Modulation of Experimental Polar Surface Area (EPSA) in molecules

Dynamic physicochemical polarity descriptors like radius of gyration (R_g_), EPSA, IMHB, etc., Must be considered to quantify chameleonic scores in molecules. Chameleonic scaffold design must include an extended polar surface area between the aqueous and lipidic phase for better therapeutic efficiency (Linker et al., 2022).

#### 5.4.4 Chemical Modification through N-methylation

Selective N-methylation in amide molecules can induce reduce hydrogen-bond donor counts making the molecules more compact in non–polar environments inducing chameleonicity. An equilibrium between aqueous solubility and membrane permeability is the primary objective behind such chemical modification in chameleonic molecules (Ramelot et al., 2023).

#### 5.4.5 Computation and AI-based design approaches

Computational Molecular dynamics (MD) simulations clubbed with Machine Learning (ML) strategies can effectively design such cyclic peptides with chameleonic properties. Quantitative structure-activity relationship analysis adds to designing specific peptides with chameleonic properties to be employed as drugs, overcoming the bRo5 rule. Hypothetical designed structures of macrocycles, peptides, PROTACs, etc., Plausible to meet the requirements are considered as inputs. The input structures are standardized with respect to molecular weights, TPSA values through several Cheminformatics software toolkit like RDKit, PubMed (a database often referred), Epik, etc. In these software tools the SMILES, Mol2, etc., raw structures of compounds are fed to check their ionization at physiological pH, druggable parameters, TPSA, etc. Creating a conformational ensemble specially in non–polar mediums leading to modification of input SMILES structures of their test molecules. Thus, specialized conformational simulations are required to generate environment specific conformational ensembles. Mixed Monte Carlo Simulations, Torsional Sampling, Molecular Dynamics, Torsion free Search Algorithms are some worth-mentioning computational methods used in generating such ensembles. A multi-stage computational workflow is required for molecular parameterization, topology generation, system construction, energy minimization, equilibration, and production dynamics (Poongavanam et al., 2018; Tang et al., 2023). A comprehensive computational analysis integrates multitude of molecular physicochemical descriptors needful to assess chameleonicity.

**FIGURE 10:**
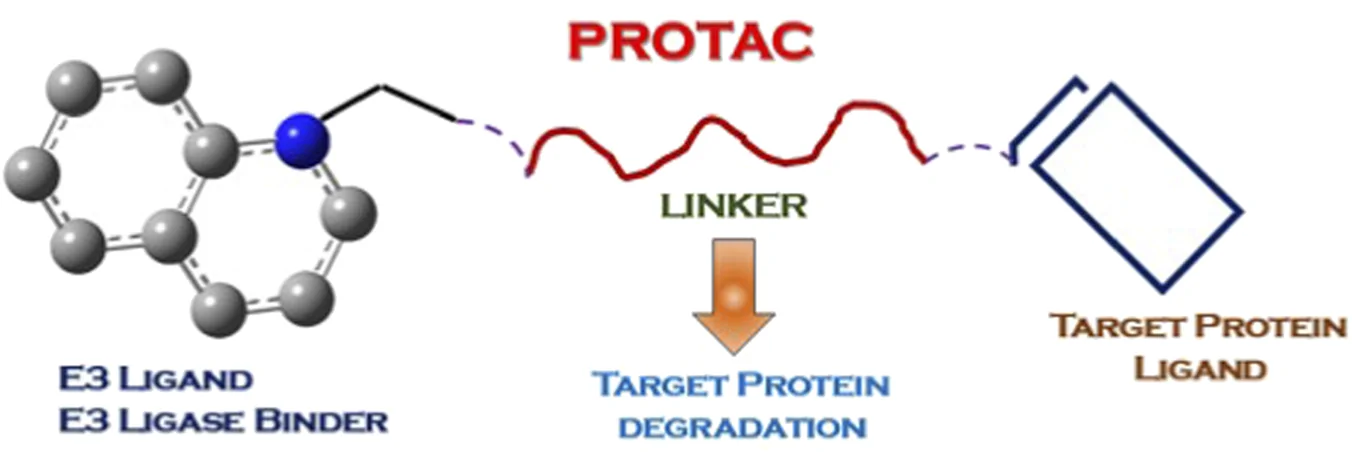
Schematic diagram of a PROTAC.

Here, PROTACs are the notable ones to mention. PROTACs are high molecular weight non-conventional molecules with chameleonic properties possessing significant cellular permeability. These molecules can dynamically reorient themselves in non-polar environments achieving a folded conformation. The schematic diagram of a PROTAC can be visualized in Figure 10. Hornberger et al. (Hornberger and Araujo, 2023) discusses the optimization of orally bioavailable PROTACs with improved bioavailability. The standard physicochemical descriptors may be listed as.

- Hydrogen Bond Donors (HBD): ≤2 unsatisfied HBD
- Hydrogen Bond Acceptors (HBA): ≥15
- Molecular Weight: optimized up to 950 Da
- Topological Polar Surface Area (TPSA): ≤200 Å^2^
- Rotatable Bonds (RB): ≤14 bonds
- Aromatic Rings: ≤4
- Lipophilicity (cLogP/cLogD): cLogP ∼1 – 7 and cLogD ∼0 to 6

The solvent dependent chameleonic behavior of PROTACs can be engineered through the *linker* design. The polar surface area of such molecules is effectively decreased; the major part being played by the linker which masks the polar functionalities promoting the formation of intramolecular hydrogen bonding (IMHB). This altogether leverages passive diffusion through the membranes to a considerable extent. The rigidity as well as the flexibility of the linker is the key to design effective PROTACs. Conformationally adaptable linkers in comparison to highly polar and flexible linkers exhibit enhanced cellular permeability (Testa et al., 2018).

Vepdegestrant (ARV-471) is the first FDA approved PROTAC to reach the market. It is a heterobifunctional targeted protein degrader to selectively target and degrade the Estrogen Receptor (ER) in ER+/HER2-breast cancer patients. PROTAC design and development is essentially a druggable proteome system beyond the rule of 5. Huang et al. showed the development of a novel SIRT6 degrader–SZU-B6, which induced its complete degradation in SK-HEP-1 and Huh-7 cell lines and significantly inhibited hepatocellular carcinoma (HCC) cell proliferation than the parental inhibitors. Thus, this inhibition mechanism points towards the efficient PROTAC strategy to adopt conformational stability while preserving its stable spatial orientation. The SZU-B6 inhibitor enhanced the cellular radiosensitization promoting tumour cell damage in hepatocellular carcinoma (Huang et al., 2024). Development of CDK9 inhibitors is extremely challenging owing to similarities in its ATP binding sites. Qiu et al. reported a series of PROTACs developed from CDK9 inhibitor BAY-1143572. Among these B03 emerged out as a leading CDK9 degrader even at nanoscale concentrations with superior antiproliferative activity compared to parent inhibitors. This is a leading example of conformational adaptation to enhance membrane permeability mitigating the importance of chameleonicity in PROTAC based molecules (Qiu et al., 2021). A subtle balance between chameleonicity and safety assessment is another promising research Frontier to be considered while designing and developing PROTAC based drugs. A critical challenge in PROTAC development is degradation of neosubstrates. PROTACs induce the degradation of therapeutic targets exploiting the natural substrate-recognition machinery of cereblon. Similar mechanistic approaches promote the degradation of non-targeted endosomal proteins. Thus, optimization of PROTACs in addition to addressing increased pharmacokinetic ability must also consider the broader degradational picture (Garside et al., 2026).

**FIGURE 11:**
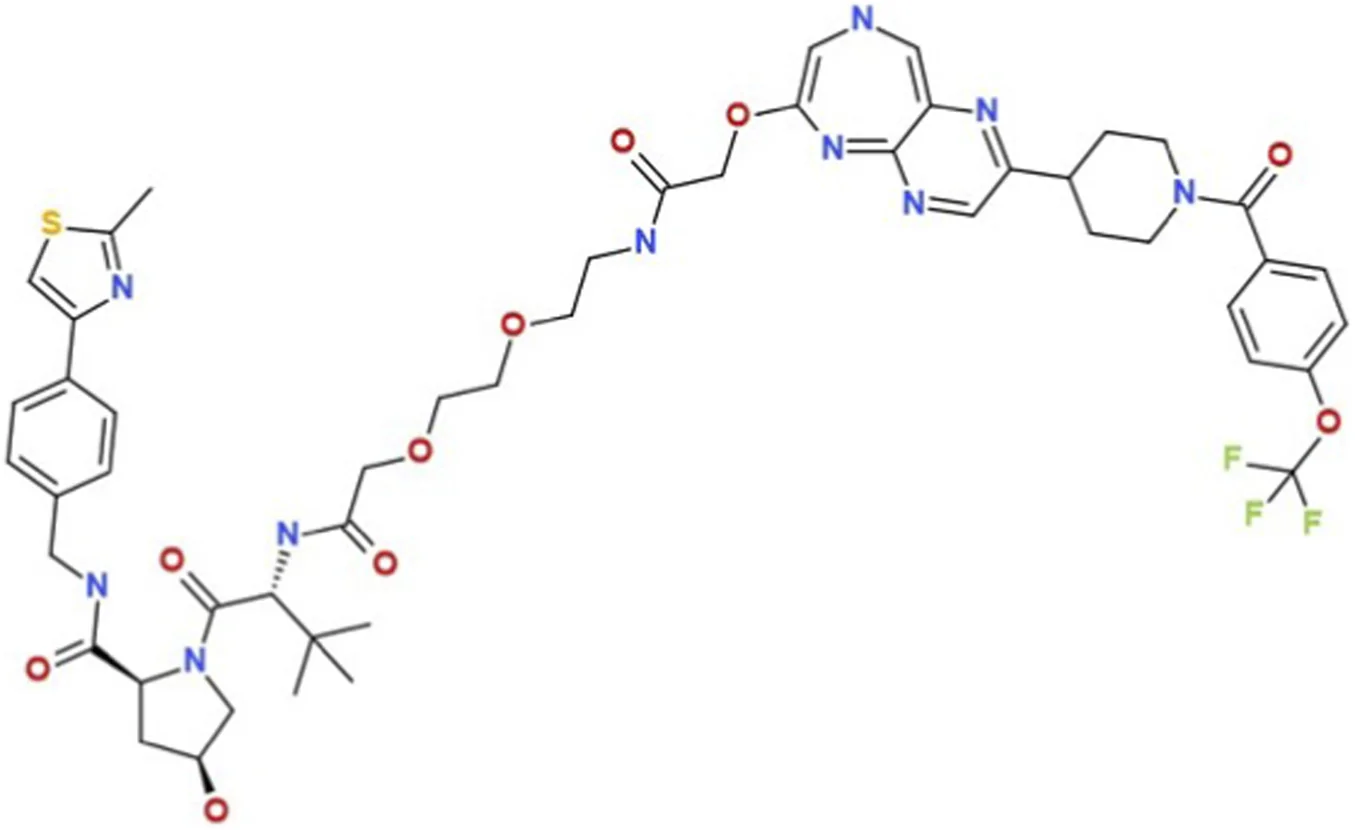
Chemical structure of PROTAC 1.

Atilaw et al. depicted the increased cell permeability in PROTAC 1 (Figure 11). PROTAC 1 exhibits chameleonic properties, characterized by a neutral pH value and high proportions of folded conformations in nonpolar environments, in contrast to linear structures in polar environments (Atilaw et al., 2021).

Ermondi et al. employed this molecule to predict the conformational descriptors of chameleonic PROTACS. The effectiveness of conformational sampling (CS) protocols and schemes in predicting and accurately reproducing the experimental results from NMR-based structural conformations and physicochemical data was portrayed in their article (Ermondi et al., 2023). This type of approach was strategized in other literary articles too (Jorgensen et al., 1996; Stewart, 2013). Conformational strategies in correlation with experimental data, though not comprehensively with detailed protocols, were suggested through their article. They routed the SAR in such a way that it minimized the time and cost for detailed experimental analysis. This computational strategy is a sustainable one, reducing the chemical pathways and paving the groundwork for targeted chemical synthesis.

The simulation protocols summarized in Table 7 are some contemporary tools for evaluating chameleonic behaviour though their design strategies mainly rely on conformational flexibility, intramolecular hydrogen bonding, macrocyclization, N-methylation, linker design, etc. Thus, the sampling strategies discussed are basic optimization protocols rather than design strategies.

**TABLE 7:** Substantiative overview of the methodologies employed for conformational sampling (Ermondi et al., 2023).

Software employed	Protocol label	Algorithm	Parameters	Force field	Solvation model	Current trends
Protocols for Conformational Sampling (CS)						
Schrodinger	Sc01	Mixed torsional/low-mode sampling. LMOD	Maximum number of steps = 10^3^	OPLS; Generalized-born/Surface-area	(GB/SA) model	Small molecule conformer generation
Schrodinger	Sc02	Mixed torsional/low-mode sampling. LMOD	Maximum number of steps = 10^4^	OPLS; Generalized-born/Surface-area	GB/SA model	Flexible molecule conformer generation
Schrodinger	Sc03	Mixed torsional/low-mode sampling. LMOD	Maximum number of steps = 10^5^	OPLS; Generalized-born/Surface-area	GB/SA	Comprehensive sampling for chameleonic molecules
Schrodinger	Sc04	Torsional sampling (MCMM)	Maximum number of steps = 10^3^	OPLS; Generalized-born/Surface-area	GB/SA	Rigid molecule conformer generation
Schrodinger	Sc05	Torsional sampling (MCMM)	Maximum number of steps = 10^4^	OPLS; Generalized-born/Surface-area	GB/SA	Exhaustive sampling for flexible conformer generation
Schrodinger	Sc06	Torsional sampling (MCMM)	Maximum number of steps = 10^5^	OPLS; Generalized-born/Surface-area	GB/SA	Extensively used for biomolecules like protein – ligand systems

Software	Method label	Starting conformations	Parameters	Hamiltonian	Solvation model	Current trends
Protocols for post-computational sampling-minimization						
MOPAC	MO1	Clusters from Sc01	Default	PM7	COSMO	Semiemperical, fast
Gaussian 16	G16 – Opt	Clusters from any source	DFT (B3LYP-D3BJ)	Full quantum mechanical (QM)	SC-PCM	Structure - property prediction, accurate

Zhou and Huang highlighted in their review article the integration of computational modeling and machine learning methods in enzyme design (Zhou and Huang, 2024). The complex enzyme design, coupled with machine learning methodologies, introduces advancements like AlphaFold2, which facilitates structure-based enzyme design in 3D databases. Emphasis is placed on predicting machine learning techniques for designing efficient molecules. Thalmayr et al. discuss screening multiple topologies for molecular chameleon carriers that combine lipoplexes and polyplexes with tunable properties and architecture for mRNA therapeutics and gene therapy (Thalmayr et al., 2023). Bai et al. developed a “burn-after-reading” approach for polyurethane films that enables multidimensional, dynamic data encryption (Bai et al., 2025). These SAR protocols may be diversified for designing other PROTACS, too, with targeted binding capacity and increased drug efficacy.

Several ML architectures including Random Forest (RF) and Support Vector Machine (SVM) models are useful in predicting *in-vitro* ADME properties from the physicochemical descriptors for bRo5 compounds. Currently, Graphical Neural Networks (GNN) and deep learning architectures are gaining prominence owing to their efficiency in predicting topological features and molecular level interactions (Elton et al., 2019; Stokes et al., 2020). AIML models can be trained to extract out the data for chameleonic molecules from the huge repositories like ChemBL, macrocyclic libraries, drug databases, Macrocycle-DB etc. Successful integration of such data based chameleonic parameters with experimentally determined values can assist in identifying chameleonic behaviour in certain molecular architectures with adaptive polarity conformations (Kihlberg et al., 2021; Jiang et al., 2026; Hunter et al., 2025).

**FIGURE 12:**
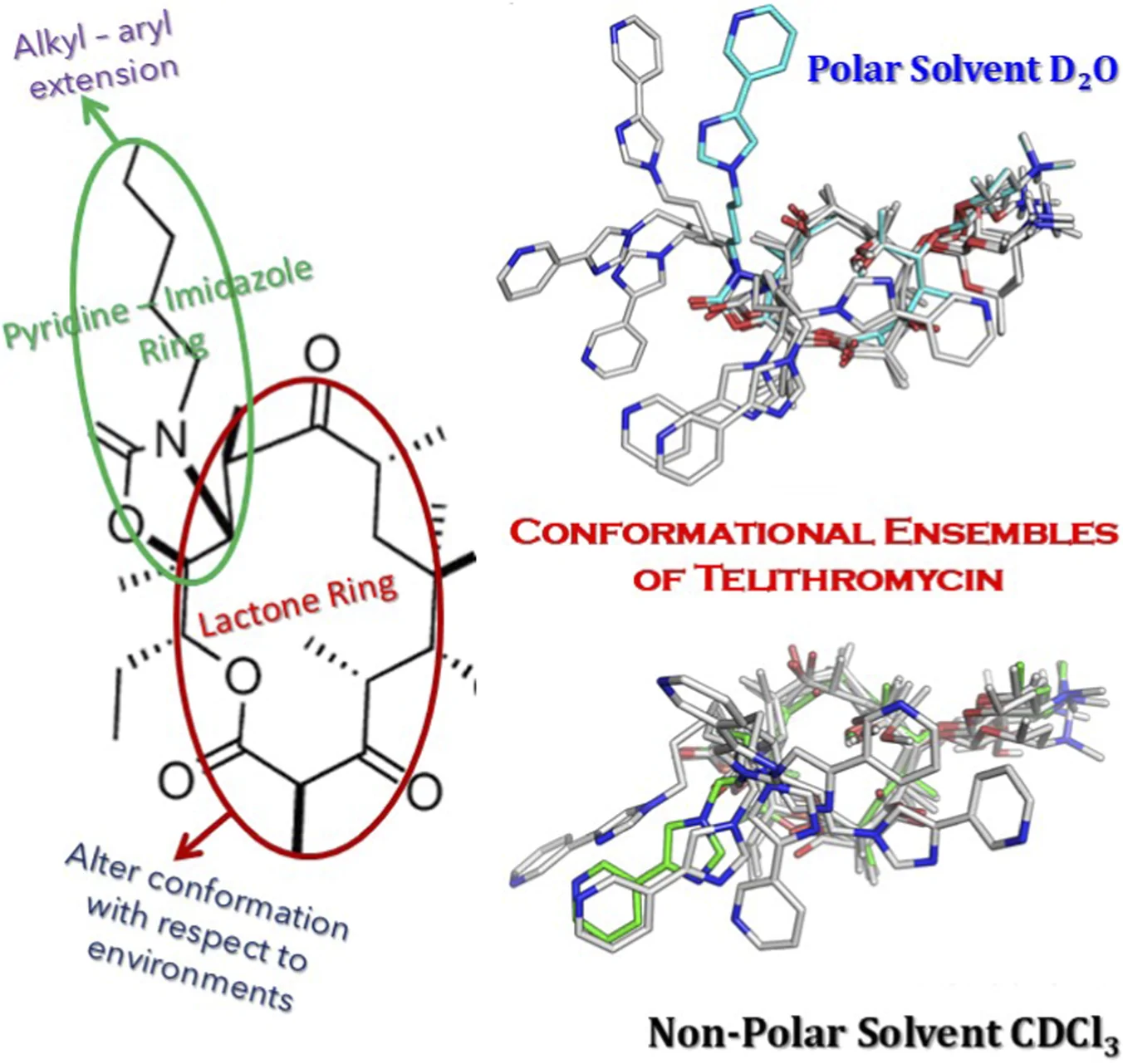
Representative conformational ensemble of the chameleonic drug Telithromycin in polar (D_2_O) and non-polar (CDCl_3_) solvents. (Reprinted (adapted) with permission from Chemistry: A European Journal, 2020, 26, 5231−5244. Copyright 2020, John Wiley and Sons and Copyright Clearance Center”) (Danelius et al., 2020).

An ensemble of the chameleonic molecule Telithromycin in polar D_2_O and non-polar CDCl_3_ solvents as revealed in the articles (Poongavanam et al., 2024 and Danielius et al., 2020) may be represented by Figure 12.

## 6 The entropy penalty in molecular chameleon design

Molecular chameleons exhibit a significant number of conformational states owing to their structural flexibility which in turn induces “conformational freedom” in these molecules, i.e., possessing high entropy or disorderliness. In scientific terms these molecules have substantial conformational entropy with extreme degree of disorder. Thus, deploying such conformationally disordered molecules in therapeutic terms is cumbersome and requires momentous design specificity to overcome this high ΔS. Thus, the key strategies devised by scientists is pre-organization of these molecular chameleons into restrictive conformations reducing the number of flexible structures it can flip-flop (He et al., 2024). Pre-organization via macrocyclization reduces the conformational space available to such molecules reducing the numbers of flexible orientation. Thus, a trade-off between their flexible conformations possessing high degree of disorderliness along with increased permeability to a decrease in its binding affinity and bioavailability was discussed by Doak *et. al* in his article (Doak Bradley et al., 2014). Steadman et al., in his article demonstrated Cyclophilin inhibitors derived from Sanglifehrin A to show this classic trade-off between thermodynamic and structural parameters in chameleonic molecules. Initially synthesis of Cyclophilin inhibitors, targeted for treatment of hepatitis C required an impractical ∼40 step synthesis procedure. But, macrocyclic Cyclophilin compounds derived from the natural product Sanglifehrin A aided to overcome these synthetic challenges. Structural optimization identified the valine-m-tyrosine-piperazic acid tripeptide of Sanglifehrin A to maintain the rigidity with robust carbon stereo-centres and free hydroxyl group of the m-tyrosine residue, thus rescuing the thermodynamic cost. This was further mitigated to replacing the flexible diene moiety of the macrocycle by a rigid styryl group which introduced stable π stacking interactions with Arg55 and Cyclophilin A. Thus, this conformational restriction reduced the entropic penalty while maintaining the molecular flexibility enabling the synthesis of potent drug candidates with improved oral bioavailability (Steadman et al., 2017). Chang et al. highlighted the need to design a flexible molecule with some degree of rigidity to bind with specific targets without compensating a large amount of energy (Chang et al., 2007). The molecules are to be pre-organized in its rigid bioactive conformation to compensate the disorderliness in its flexible structures in order to offset high entropy. Foo et al. in their recent editorial have highlighted macrocyclization to bridge the lacunae between small drug molecules and large biologics ultimately paving the way to new generation benchmarked therapeutics (Foo et al., 2026). Designing molecular switches with N-methylated side chains which adjust their structures with respect to the environmental conditions underscoring their binding proficiency negate the rise of entropy while binding with targeted substrates. ΔPSA quantifies chameleonicity in a molecule which ranges around ∼0.2 × Molecular Weight – 140 Å^2^ for an effective molecular chameleon design (Poongavanam et al., 2024). *Chamelogk* was introduced by Jimenez et al. as a computationally efficient and user-friendly dynamic descriptor of chameleonicity. It is a quantitative chromatographic chameleonic descriptor to design orally bioavailable chameleonic drugs integrating polarity and lipophilicity parameters. The underlying difference between intrinsic and experimentally determined polarity is attributed to the physicochemical basis of determining *ChamelogK*. It accounts for the physicochemical deviations arising from the linear trend, enabling a comparison between different conformational forms. A threshold *ChamelogK* value of 0.6 is generally taken as an alert value for chameleonic molecules (Jiménez et al., 2023). Incorporation of intramolecular interactions like hydrogen bonding, etc., can also strike a balance between molecular flexibility and thermodynamic constraints leading to increased entropy.

## 7 Potential drawbacks of molecular chameleons

Molecular chameleons, though, present several advantages in regards to their alternating conformations and adaptability to environmental changes, projecting a greater potential for drug discovery, albeit with certain drawbacks. The prime hurdle in designing a molecular chameleon is their structural complexity, macrocyclization constraints, and multiple stereocenters (Latosińska and Latosińska, 2024; Tyagi et al., 2018; Wieske et al., 2023). The high flexibility offered by molecular chameleons makes their synthesis quite challenging and requires advanced synthetic techniques for their development and production. Late-Stage Functionalization (LSF) is a powerful tool which strategizes the modulation of physicochemical descriptors without constructing the whole molecular architecture. Improvising synthetic strategies to modify substitutions/functionalization, altering hydrogen bonding donor-acceptor moieties, induce controlled steric hindrance and introducing hydrophobic moieties for better conformational adjustments in such molecules are the primary attributes of the LSF strategy. N-methylation synthetic LSF strategies are capable of reducing exposed IMHBs, facilitating polarity masking and inducing compactness in structures. Castellino et al. advocated for the LSF techniques as modern tools for enhancing druglike properties. To reduce synthetic efforts through LSF and to modify complex molecules towards the later stages of the synthetic procedure, thus reducing time and enhancing the yield of multistep synthesis, are the main highlights of the article (Castellino et al., 2023). Thus, LSF strategies is capable of addressing the synthetic and structural challenges associated with chameleonic behaviour in polarity sensitive environments. Another challenge posed by molecular chameleons is their high conformational flexibility, which makes their functional predictability uncertain. The dynamic nature of such molecules, along with their adaptability to multiple environmental conditions, makes their pharmacokinetic and pharmacodynamic modeling complicated (Hornberger and Araujo, 2023). Complexity in molecular characterizations, off-target interactions, manufacturing loads, and stringent regulatory protocols are some other drawbacks faced by the design, development, and implementation of molecular chameleons in drug discovery. Co-existence of multiple conformational forms that dynamically switch in between due to changes in environmental polarity, pH, temperature, etc. This conformational heterogeneity is often not addressed by the regulatory protocols. Different conformations have different physicochemical characteristics and pose significant problems in batch productions. A defined set of Critical Quality Attributes (CQA) for molecular chameleons is a *misnomer* and are often not confined to traditional parameters (% purity, assay results, impurity profiles). High end instrumental validation (NOESY/ROESY, mass spectrometry, hydrogen–deuterium exchange methods, etc.) is often required to integrate Chemistry-Manufacturing-Controls (CMC) with their pharmacokinetic/pharmacodynamic performance (Ermondi et al., 2023). Conventional *‘Quantitative Structure Activity Relationship’* (QSAR) protocols are largely limited in predicting the structure-activities of chameleonic molecules owing to their dynamicity in their conformations and behaviour. It is important to note that static descriptors like molecular weight, lipophilicity, TPSA, IMHBs generated from a single optimized molecular geometry is insufficient to predict the structure–activity relationship in chameleonic molecules. Understanding and assessing dynamic physicochemical descriptors for molecular chameleons, e.g., R_g_, EPSA, SASA, etc., requires comprehensive ensemble generation as discussed in Section 4.4 from a large number of samples may be beneficial in addressing the current limitations of classical QSAR methodologies. An integrated computational-AI/ML based approach for dynamic modelling of the ensembles generated will generate better physicochemical descriptors required for effective *‘molecular chameleon’* design (Jimenez et al., 2024b; Ermondi et al., 2023). Ermondi et al. (Ermondi et al., 2023),, Tang et al. (2023) and Polishchuk et al. (2013) highlighted the characterization, synthetic, and modeling drawbacks of these molecules in their articles.

## 8 Summary

In a nutshell, molecular chameleons are the most coveted molecules in the modern scientific genre, with a plethora of contrasting and flexible characteristics that allow them to adjust in both polar and nonpolar environments. Dynamic physicochemical descriptors as Experimental Polar Surface Area (EPSA), quantification of IMHBs, conformational ensemble analysis, assessment of polarity sensitive environmental parameters are the new paradigms to evaluate chameleonicity in a molecule in addition to static parameters like molecular weight, cLogP, TPSA, etc. The segmental flexibility in these molecules is achieved through their structural modifications and design improvisations, which enable them to be tailored for biologically benchmarked applications. The limitations encountered by large molecules in inducing drug properties can be addressed by functional molecules that fall outside the Bro5, which can be modulated to leverage chameleonic properties and overcome such shortcomings. The chemistry behind the design and synthesis of these types of molecular chameleons, particularly cyclic macrocycles, cyclic peptides, PROTACs, etc., is inscribed in this article, where increased bioavailability descriptors are taken into account. Clinical milestones achieved include the FDA approval of the PROTAC, Vepdegestrant (ARV-471), advancement of macrocyclic oral drug enlicitide (MK0616) etc. Computational simulations and SAR investigations also prove to be sustainable in such chameleonic molecular design. The review highlights modern methodologies and tools based on chemical and computational aspects for designing molecular chameleons. This approach includes strategic engineering, chemical modification, scaffold re-modelling and AI based ensemble prediction, optimization and manipulation for better therapeutic control.

## 9 Future perspectives

The future of molecular chameleons from the perspective of modern research landscape is quite promising. These molecules offer adaptive and responsive solutions in drug delivery and bioavailability and are often considered smart therapeutic solutions for various diseases. The development of nucleic acid-based drug delivery vehicles and the integration of artificial intelligence (AI) with such flexible molecular entities will advance the pharmaceutical domain to new heights. AI-driven approaches promote the pathway for revolutionizing drug discovery by designing molecules with a high level of structural and functional flexibility. Beyond the identification of prospective chameleonic molecules future developments in this genre must aim to address the *“Chameleon Paradox”.* Apart from the flexibility aspects to improve bioavailability for bRo5 drugs, care must be taken to ensure the limiting rise of entropy which eventually decreases the targeted drug–receptor binding. AI based approaches must leverage generation of predictive models encompassing molecule adaptability, targeted binding, entropic limitations and improved bioavailability rather than limiting to screening the respective databases. Dynamic Combinatorial Chemistry (DCC) is deployed to harness molecules with chameleonic properties that can easily adapt to the structural requirements for targeted binding, interactions, enhancing specificity and efficacy through generation of predictive chameleonic architectures taking all the intended functionalities into consideration.

Computational approaches with generation of appropriate ensembles specially in non-polar media will be beneficial to mitigate the current limitations though thorough research on generating more samples and validating the experimental and theoretical data. Thus, integrating DCC, computational methodologies and AI can help to mitigate the current challenges in scarcity of samples, dynamic ensemble generation and experiment-theory validation.
